# Conceptualizations of compassion in the context of dementia caregiving: A scoping review protocol

**DOI:** 10.1371/journal.pone.0357411

**Published:** 2026-09-18

**Authors:** Kristina M. Kokorelias, Robert Simpson, Ron Beleno, Jill Cameron, Erica Dove, Kerry Kuluski, Carrie McAiney, Sarah E. P. Munce, Kieran Quinn, Marianne Saragosa, Marina Wasilewski, Marie-Lee Yous, Lynn Zhu, Jenette Wu, Nathan Stall

**Affiliations:** 1 Division of General Internal Medicine and Geriatrics, Sinai Health and the University Health Network, Canada; 2 Rehabilitation Sciences Institute, Temerty Faculty of Medicine, University of Toronto, Toronto, Ontario, Canada; 3 Department of Occupational Sciences and Occupational Therapy, Temerty Faculty of Medicine, University of Toronto, Toronto, Ontario, Canada; 4 KITE Research Institute, University Health Network, Toronto, Ontario, Canada; 5 Institute for Better Health, Trillium Health Partners, Mississauga, Ontario, Canada; 6 Institute of Health Policy, Management and Evaluation, University of Toronto, Toronto, Ontario, Canada; 7 University of Waterloo, Waterloo, Ontario, Canada; 8 Schlegel-UW Research Institute for Aging, Waterloo, Ontario, Canada; 9 Holland Bloorview Kids Rehabilitation, Bloorview Research Institute, Toronto, Ontario, Canada; 10 Temmy Latner Center for Palliative Care, Sinai Health, Toronto, Ontario, Canada; 11 Department of Medicine, Temerty Faculty of Medicine, University of Toronto, Toronto, Ontario, Canada; 12 Science of Care Institute, Sinai Health, Toronto, Ontario, Canada; 13 John’s Rehab, Sunnybrook Research Institute, Toronto, Ontario, Canada; 14 School of Nursing, McMaster University, Hamilton, Ontario, Canada; 15 Ontario Hospital Association, Toronto, Ontario, Canada; 16 Ontario Tech University, Oshawa, Ontario, Canada; Essen University Medical School, GERMANY

## Abstract

**Background:**

Compassion is widely recognized as a vital component of dementia caregiving, yet its conceptualization, definition, and operationalization remain inconsistent across contexts. In dementia caregiving, compassion is often conflated with empathy, emotional labor, and relational care, obscuring its unique characteristics and applications. Clarifying how compassion is understood and applied, particularly among unpaid family caregivers, is essential for advancing research, policy, and practice.

**Objective:**

This paper outlines a scoping review protocol to map and synthesize empirical studies examining the conceptualizations, definitions, and operationalization of compassion in dementia caregiving by unpaid family caregivers. The forthcoming review will identify how compassion is framed and measured across diverse care settings and caregiver populations, providing a foundation to inform future compassion-based interventions and assessment tools.

**Methods:**

Following Joanna Briggs Institute (JBI) methodology and the PRISMA-ScR reporting guidelines, this forthcoming review will systematically search CINAHL, EMBASE, MEDLINE, and PsycINFO. Eligible studies include qualitative, quantitative, and mixed-method empirical research focused on family caregivers of persons living with dementia. Studies exploring compassion or self-compassion conceptualization, definition, or operationalization will be included. Non-empirical works, reviews, and paid caregiving roles are excluded. Screening and data extraction will be conducted independently by two reviewers using a form informed by the Compassion-Focused Care Framework (CFCF), which emphasizes Affiliative relationships, Bidirectional communication, Compassionate partnerships, and the structural supports of Compassionate Design, Education, and systemic Call to Action.

Data will be synthesized using descriptive statistics and qualitative content analysis.

**Patient and Public Involvement:**

Caregiver partners from Alzheimer’s Societies and Ontario Health Teams will contribute throughout the review process, including study selection, interpretation, and dissemination. Engagement is structured according to the Ontario Brain Institute framework and CIHR principles, ensuring inclusiveness, mutual respect, support, and co-building. A peer-researcher with lived caregiving experience will lead patient engagement efforts.

**Discussion:**

This review will provide a comprehensive synthesis of how compassion is understood and applied in family dementia caregiving, addressing a critical gap in the literature. By identifying existing measures and conceptual frameworks, the findings will inform the development of more nuanced, caregiver-centered interventions and policies aimed at enhancing compassionate care. Ultimately, this work seeks to support caregivers’ well-being and improve care quality for people living with dementia.

## Introduction

Compassion is a fundamental aspect of paid and unpaid caregiving [1], particularly in the context of dementia, where caregivers often face complex and multifaceted challenges that can result in compassion fatigue [2,3]. Compassion is commonly understood in the broader literature as a multidimensional response that involves recognizing the suffering of others, experiencing an emotional resonance, and being motivated to alleviate that suffering through action [4]. Compassion also involves a dedication to understanding the suffering of others, coupled with an unwavering commitment to alleviate it [5]. It extends beyond empathy by incorporating a desire to help, and it is considered a key component of ethical and relational care across various disciplines, including health, psychology, and philosophy [6]. In the context of caregiving, especially for persons living with dementia (PLWD), compassion plays a critical role in shaping how care is experienced and delivered [7]. Compassion in this context serves not only as a motivator for providing patient-centered care but also as a protective factor that can help mitigate caregiver burnout by fostering a sense of meaning and connection in the caregiving relationship [7].

The majority of caregivers to persons living with dementia are family members or close individuals who provide unpaid care [8]. For caregivers of persons living with dementia, compassion is especially critical because the progressive nature of dementia can intensify both the physical and emotional demands placed on caregivers, often leading to high levels of stress and compassion fatigue [2]. Compassion fatigue is a form of emotional, physical, and psychological exhaustion that can occur in caregivers after prolonged or repeated exposure to others’ suffering [9]. It is associated with a reduced ability to engage empathetically or maintain the same level of compassionate care [9]. Dementia caregiving requires addressing not only the physical needs of persons living with dementia but also their emotional, psychological, and social well-being [10,11]. At the same time, caregivers must navigate the profound impacts that caregiving can have on their own physical, mental, social, and financial health [12]. These dual demands (i.e., meeting the complex needs of the person living with dementia while managing personal strain) underscore the importance of understanding and supporting compassion in caregiving relationships. Compassion has been identified as a critical factor in alleviating caregiver burden, fostering caregiver resilience, and improving the overall quality of care [11,13,14]. The Compassionate Family Care Framework (CFCF) is an evolving approach that seeks to integrate compassionate care practices into family caregiving, particularly in healthcare settings [15]. This framework aims to better understand how compassionate care can be delivered not only to the person receiving care, but also to the family unit as a whole [15]. While the CFCF framework is well-developed and has been applied primarily in neonatal and pediatric populations, there is a lack of understanding of how its principles translate to dementia caregiving, where caregiver distress and compassion fatigue present unique challenges, highlighting the need for our focused review. Despite its recognized importance, the concept of compassion in dementia caregiving remains variably defined and understood, limiting its systematic integration into caregiving practices, training, and policy development.

To advance the understanding and application of compassion in dementia caregiving, it is essential to clarify how the term is conceptualized (i.e., how a concept is *understood or framed)*, defined (i.e., *making the meaning of a term explicit)* and operationalized (i.e., how that defined concept is *measured or applied*) across various contexts (e.g., caregiving at home, caregiving in long-term care homes, gender differences). Existing research on compassion spans multiple disciplines [4,16], encompassing perspectives from healthcare [17,18], psychology [19], and ethics [20]. In dementia caregiving, compassion is often conflated with empathy [21], emotional labor [22], and relational care [23], but its unique attributes and applications remain underexplored. Without clear definitions and measures, efforts to foster compassion may lack consistency, potentially diluting their effectiveness and scalability. Qualitative studies, which provide rich, in-depth insights into lived experiences and contextual factors [24], are particularly well-suited for exploring the nuanced conceptualizations of compassion. However, the breadth and scope of such studies have not been rigorously reviewed to date.

This protocol outlines the methodological approach for a scoping review of the literature aimed at mapping and synthesizing empirical studies on the conceptualizations, definitions, and operationalization of compassion in dementia caregiving. Thus, this review will answer “How are compassion and self-compassion conceptualized, defined, and operationalized in dementia caregiving by unpaid family caregivers, and what outcomes are associated with their presence or absence?” For this review, we will specifically include unpaid family caregivers, while excluding paid caregiving roles. By identifying and analyzing how compassion is understood and experienced in this context, the review will provide a comprehensive foundation for advancing research, policy, and practice. It may also help identify which measures of compassion have been used, offering insight into their sensitivity and limitations. This could inform future efforts to adapt or co-develop measures of compassion that are meaningful and applicable to the lived experiences of family caregivers.

## Methods

### Study design

This scoping review will be conducted following the JBI (formerly Joanna Briggs Institute) methodology for scoping reviews [25] and will be guided by an a priori protocol registered in the Open Science Framework (osf.io/r734a). The review will be reported following the Preferred Reporting Items for Systematic Reviews and Meta-Analyses extension for scoping reviews (PRISMA-ScR) [26]. The protocol was informed by the PRISMA for systematic review protocols (PRISMA-P) [27] (see Supplemental **Material A**). PRISMA-P provides a comprehensive framework for protocol development, that helps enhance clarity and reproducibility, even when adapted for scoping review methods [26]. This study has not yet begun.

### Conceptual framework

**Fig 1:**
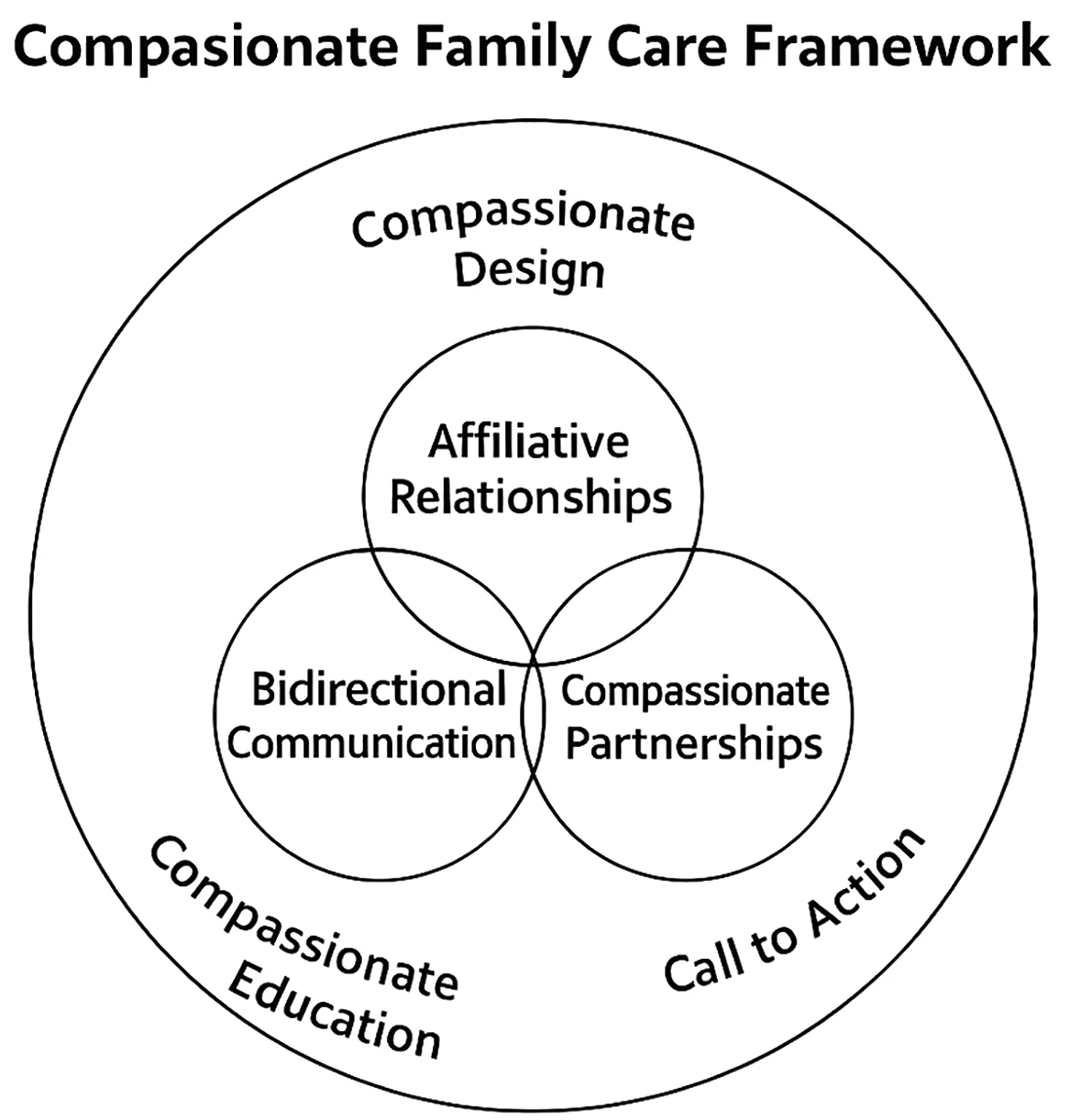
CFCF framework.

The CFCF framework considers multiple domains of compassion, including emotional responsiveness, relational understanding, and practical support, and encourages the recognition of caregivers’ own suffering and need for acknowledgment, inclusion, and care (Fig 1). The CFCF highlights how caregivers interpret acts of compassion (or lack thereof) within the health system and in their own caregiving roles, and how this shapes their ability to provide sustained, empathic care. The framework is organized around three foundational elements, referred to as the “ABCs” of compassionate care: Affiliative relationships, Bidirectional communication, and Compassionate partnerships. Affiliative relationships focus on building respectful, emotionally attuned bonds between caregivers, families, and infants. Bidirectional communication ensures that dialogue flows both ways, centering mutual understanding and trust [15]. Compassionate partnerships promote shared decision-making and recognize families as active members of the care team [15].

The CFCF also recognizes that compassion must be supported structurally and systemically through compassionate design, education, and leadership (call to action) [15]. Compassionate Design focuses on creating care environments and organizational systems that support compassionate interactions by ensuring physical spaces, workflows, and policies reduce stress and promote emotional safety for both patients and caregivers [15]. Compassionate Education equips healthcare professionals with the skills, knowledge, and attitudes necessary to provide empathetic, respectful care, emphasizing communication, emotional awareness, and self-compassion as teachable and vital competencies [15]. Self-compassion is defined as extending kindness, understanding, and support to oneself in times of difficulty [28]. The Call to Action urges healthcare leaders, organizations, and policymakers to embed compassion as a core value in all aspects of care delivery, advocating for systemic commitment through supportive policies, leadership engagement, resource allocation, and partnership with patients and families [15]. These elements involve creating supportive care environments, investing in training that builds relational skills, and fostering a culture where compassion is modeled and valued by leaders [15]. These components work together to foster an inclusive approach to compassionate care that recognizes and supports the entire caregiving unit [15]. Although initially developed more broadly, to the best of our knowledge, this framework has not yet been applied in the context of dementia caregiving-a context where caregiver distress and compassion fatigue are particularly pronounced [15]. This broader understanding can serve as the basis to identify strategies for fostering compassion in both healthcare providers and family caregivers, ultimately improving outcomes for patients and their families [15].

In this review, the CFCF will guide our data extraction and analysis by helping us identify how compassion is conceptualized, defined, and operationalized in the context of family caregiving for persons living with dementia. This theoretical grounding will also support the identification of gaps in how compassion is measured and experienced, potentially informing the future development or adaptation of compassion-related interventions or assessment tools.

### Search strategy

The search strategy for this review will be developed using the three-step search strategy recommended by JBI for scoping reviews [25]. Initially, a pilot search of the MEDLINE database was conducted by the first author prior to protocol development. The search strategy combined keywords and subject headings related to “compassion,” “family caregiving,” “dementia” and “empathy.” The first 50 papers from the pilot search were analyzed to explore text words that relate to “compassion” in the titles and abstracts of the identified papers, alongside the respective descriptive index terms. This step was being used to inform a search strategy developed by an information specialist (CDC) incorporating all identified keywords and index terms. This search will be peer-reviewed by a second information specialist in accordance with the Peer Review of Electronic Search Strategies (PRESS) guidelines [29]. This step also helped to inform the eligibility study.

This final search will be performed in CINAHL, EMBASE, MEDLINE, and PsycINFO in late October 2025. Finally, two members of the research team will screen the reference lists of all included studies to identify additional relevant studies. The full electronic search strategy will be presented with the study results.

During the screening, the reference lists of any literature reviews (scoping or systematic) that appear from the search and may be relevant to the topic will be screened to identify additional studies that may meet the inclusion criteria.

### Eligibility criteria

This scoping review will apply specific inclusion and exclusion, as noted below.

### Population

The review will include studies that focus on individuals involved in dementia caregiving, encompassing unpaid, family caregivers (e.g., family members and friends). Studies examining perspectives from caregivers of individuals diagnosed with any form of dementia, including Alzheimer’s disease and other related disorders, will be eligible. Studies exploring these caregivers’ perspectives or experiences will be included, even if other caregiver groups are represented (e.g., caregivers to individuals with other conditions). To ensure consistency during data extraction, we will include only studies in which at least 50% of the sample are dementia caregivers or where dementia-specific data can be extracted separately.

### Concept

This review will examine evidence sources that describe how compassion, including self-compassion are conceptualized (including both predefined and emerging definitions), how these concepts are applied in dementia caregiving, and the outcomes linked to their presence or absence in caregiving experience. This is informed by previous review done by members of the research team [30,31]. To enhance clarity and reduce ambiguity during screening, we have operationalized each element as follows:

- **Conceptualization:** Studies that describe *how compassion or self-compassion is framed or understood*, such as an emotional process, a relational dynamic, an ethical principle, or a caregiver characteristic. For the purposes of this review, compassion will be explicitly distinguished from related constructs such as empathy and emotional labour; studies will be included only if they explicitly refer to compassion or self-compassion, and we will note when constructs overlap or are conflated in the original study to ensure conceptual clarity during screening and analysis.
- **Definition:** Studies that provide an *explicit definition* of compassion or self-compassion. Definitions may be drawn from existing theoretical frameworks (a priori) or generated from empirical data (a posteriori).
- **Operationalization:** Studies that describe *how compassion is applied or measured in practice*, including initiatives, practices, or frameworks aimed at fostering compassion in dementia caregiving.

The review will not restrict inclusion based on specific interventions. Instead, it will consider studies that explore how compassion is defined, perceived, and operationalized within the context of dementia caregiving. To screen for conceptualization, we will include studies that describe how compassion or self-compassion is framed or understood, such as whether it is presented as an emotional process, a relational dynamic, an ethical principle, or a caregiver characteristic. To screen for definition, we will include studies that provide an explicit definition of compassion or self-compassion. These definitions may be drawn from existing theoretical frameworks (a priori) or may be generated through empirical data (a posteriori). To screen for operationalization, we will include studies that describe how compassion is applied or measured in practice. Studies examining any initiatives, practices, or frameworks related to fostering compassion in dementia caregiving will be included.

### Context

This review will focus on studies conducted in the context of family caregiving, that may include home-based care and institutional settings such as long-term care homes, hospitals, or community care environments. Studies examining cultural, social, or organizational factors influencing compassion in dementia caregiving will also be included.

### Study type

Only empirical peer-reviewed qualitative and quantitative studies will be included in the review. Eligible qualitative studies may use methodologies such as phenomenology, grounded theory, ethnography, or descriptive qualitative approaches, and methods such as interviews, focus groups, or observations. Eligible quantitative studies may include cross-sectional surveys, cohort studies, or intervention studies that quantitatively measure compassion, self-compassion, or related constructs in the context of dementia caregiving. Mixed methods studies will be included only if the qualitative or quantitative findings are reported separately and can be extracted independently. Non-empirical studies, including opinion pieces, commentaries, editorials, and theoretical papers, will be excluded. Systematic reviews, scoping reviews, and meta-analyses will also be excluded, though their reference lists may be screened for relevant primary studies.

### Publication date and language

This review will include studies published in any language. Non-English titles and abstracts will be translated using DeepL, a reliable machine translation tool, to ensure that relevant studies are not excluded due to language barriers [32]. DeepL has been shown to provide more accurate and natural translations than many other online services, including Google Translate [33]. Where possible, a second reviewer proficient in the language will verify the translation for accuracy to ensure that relevant studies are not excluded due to language barriers.

There will be no restrictions on publication date.

### Publication status

The review will include only peer-reviewed, published studies to ensure the reliability and rigor of findings. Conference abstracts, grey literature, theses and dissertations and unpublished reports will be excluded.

### Study selection

All citations and abstracts will be uploaded to Covidence systematic review software (Veritas Health Innovation, Melbourne, Australia) to facilitate the review process [34]. Duplicate entries will be identified and removed [35]. Two independent reviewers will simultaneously screen the studies for eligibility, using Covidence to ensure consistency and reliability. A pilot test will first be conducted by screening a random sample of 25 titles and abstracts by the two authors. The purpose of this pilot is to ensure consistency in the application of the inclusion and exclusion criteria. Reviewers will meet to discuss any discrepancies and refine the inclusion criteria if necessary. The goal is to achieve at least 80% consensus (and Cohen’s kappa of 0.80 is achieved [36]) between reviewers before proceeding to the title and abstract screening phase. Next, both level 1 and level 2 screening will be conducted independently and in duplicate by two reviewers. During level 1 screening, reviewers will assess titles and abstracts for eligibility using a standardized screening form. In level 2 screening, the full texts of potentially relevant studies will be retrieved and reviewed to determine final inclusion. A pilot test of the level 2 screening will be conducted on approximately 25% of the full-text articles to assess consistency, and inter-rater reliability will be calculated for included studies. Any disagreements between reviewers will be resolved through discussion, and if consensus cannot be reached, a third reviewer (NMS) will be consulted to make a final decision.

Reasons for exclusion at the full text stage will be systematically documented to maintain transparency. A flowchart, in accordance with the PRISMA-ScR, will be used to show the process of citation elimination during the screening and additional searches, along with the reasons for excluding studies during the full-text screening phase [26].

### Data extraction

Before commencing the data extraction process, a pilot of ~5 included studies will be conducted to refine the extraction approach. This pilot will allow the research team to adjust the extraction form as needed. Additional training sessions for the extractors will be held, if necessary, by the first author, and pilot extractions will continue until an agreement of 80% or greater is achieved. Once this threshold is met, each full-text study will be independently extracted by two reviewers using the standardized extraction form created in Covidence. In cases where disagreements arise between the reviewers, they will engage in discussions to resolve the discrepancies. If needed, or a third reviewer (NS) will be consulted to reach a consensus.

The data extraction form will be informed by the CFCF, ensuring that key components such as Compassionate Design, Compassionate Education, and Call to Action are adequately represented [15]. The first set of data to be extracted will include basic study details. This will encompass information such as the study’s author(s), year of publication, title, country of study and setting (e.g., home care, long-term care home, hospital). Additionally, the study design will be documented, including the methodology used (e.g., phenomenology, grounded theory, ethnography, observational, experimental), data collection methods (e.g., interviews, focus groups, observations, surveys), sample size, and participant demographics such as age, gender, race, ethnicity, and caregiver role.

Next, the data extraction will focus on the conceptualizations of compassion and self-compassion as presented in the studies. This includes how compassion is conceptualized, defined or described, as well as how it is perceived and experienced by caregivers within the context of dementia caregiving. Furthermore, the operationalization of compassion will be examined, noting any barriers and facilitators identified by the study participants that impact the expression and experience of compassion in caregiving. We will then document any findings related to the CFCF [15]. This will include evidence of Compassionate Design, focusing on how the caregiving environment, whether physical, social, or organizational, influences the practice of compassion. Information on Compassionate Education (e.g., education related to compassion) will also be captured, detailing any training or educational efforts aimed at enhancing compassion among caregivers. The review will also extract data related to a Calls to Action, including policies, strategies, or initiatives designed to promote compassionate caregiving practices, including self-compassion if included in empirical literature. Finally, the review will capture outcomes related to compassion in caregiving, including the impacts on caregivers, care recipients, and the caregiving relationship. Studies that propose recommendations to enhance compassion in dementia caregiving will also be highlighted. To ensure the accuracy and completeness of the data extraction, it will be reviewed and verified by two independent team members, with discrepancies resolved through discussion or consultation with a third reviewer, if necessary.

### Data analysis

The data will be synthesized using descriptive and content analysis, following the scoping review analysis approach outlined by Pollock et al. [37]. Two independent authors will conduct the content analysis by categorizing the various conceptualizations of compassion found in the included studies, focusing on whether the evidence addresses compassion in terms of design, education, or action, as guided by the CFCF [15]. The different definitions of compassion found in the studies will be recorded, and we will count how many studies use each definition. These will then be organized in order from the most frequently to the least frequently used. We will also examine any differences in the experiences of barriers and facilitators to compassion among various family caregiver groups, such as children, spouses, and other caregivers, with attention to differences across ethnic, cultural and gender groups. While the CFCF will guide the initial categorization of data, we will also employ an inductive approach to capture themes and insights that emerge outside the framework [15]. Any findings not represented within the CFCF categories will be coded openly and incorporated into the analysis to ensure that novel or unanticipated conceptualizations of compassion are identified [15]. This approach allows the review to remain both theoretically informed and sensitive to new evidence from the literature. Study characteristics will be presented descriptively in a table.

### Patient and public engagement

This research will be guided by a steering committee made up of representatives from two of the Alzheimer’s Societies in Ontario, Canada, as well as one of the Ontario Health Teams. Two caregiver partners, including an adult male caregiver and a female caregiver of a person living with dementia, have also been identified to provide valuable insights on the caregiving experience. While the PPI sample includes one male and one female caregiver, it may not reflect other dimensions of diversity, such as racial, cultural, socioeconomic, or geographic differences. Additional efforts will be made to engage diverse caregiver voices, including those from racialized and rural communities, to further strengthen inclusivity. The caregiver partners will be compensated for their time, though they have chosen to decline authorship. These caregiver partners were actively involved in the development of this protocol and will remain engaged throughout the entire project, from the conduct of the review to the dissemination of the published findings. For example, they provided feedback on the clarity and wording of the eligibility criteria, suggested additional search terms relevant to lived caregiving experiences, and reviewed preliminary data extraction forms to ensure they captured meaningful aspects of compassion in dementia caregiving. They also contributed to shaping the focus and framing of research questions to reflect caregiver priorities, ensuring that the review addresses issues that are most relevant to those providing unpaid care. Their roles will continue to include assisting with screening studies, providing input on data interpretation, and contributing to the dissemination of findings. Their roles include assisting with screening studies, providing input on data interpretation, and contributing to the dissemination of findings. The engagement process is guided by the Ontario Brain Institute (OBI) framework to ensure meaningful and structured collaboration [38] and Canadian Institutes for Health Research’s core principles of inclusiveness, mutual respect, support, and co-building in all aspects of the work [39,40]. We will ensure *inclusiveness* by engaging diverse caregiver voices, including those from racialized and rural communities. *Mutual respect* will be upheld by valuing caregiver expertise equally alongside academic and clinical knowledge. We will provide *support* through honoraria, flexible meeting times, and accessible materials. *Co-building* will be reflected in collaborative development of study materials, interpretation of findings, and co-authorship, ensuring caregivers help shape the review at every stage.

Additionally, our peer-researcher, RB, who has personal experience as a caregiver and advocate for persons living with dementia and their caregivers, will lead our patient engagement efforts, ensuring that the voices of caregivers are central to the review process. This will include co-developing study materials such as the screening inclusion/exclusion criteria training, supporting interpretation of findings through a caregiver lens, and leading the process for co-authoring knowledge translation products to ensure caregiver perspectives are meaningfully reflected throughout the review.

### Knowledge translation

This scoping review will engage in Integrated Knowledge Translation (IKT) throughout the entire process to ensure that the findings are relevant, actionable, and directly beneficial to interest holders, particularly caregivers and healthcare professionals [41]. IKT emphasizes the collaborative approach between researchers and knowledge users to ensure that research is not only academically rigorous, but also applicable in real-world settings [41,42]. Consistent with IKT, we will maintain an ongoing dialogue with our patient partners, obtaining feedback and ensuring that the research process remains responsive to their needs and priorities. A key aspect of our IKT approach will be the creation of a concise, accessible report, specifically for our partner organizations. This report will summarize the findings of the scoping review and provide actionable recommendations that can be used to inform their ongoing work with caregivers of people with dementia. We will also hold frequent meetings with our caregiver partners to share emerging findings. Finally, the findings will be submitted for publication in a peer-reviewed journal and presented at relevant conferences.

## Discussion

The forthcoming scoping review aims to provide a comprehensive understanding of how compassion is conceptualized, defined and operationalized in the context of dementia caregiving. By synthesizing studies, we will see how the multifaceted nature of compassion in caregiving is conceptualized within qualitative studies related to dementia caregiving. Existing literature has often conflated compassion with related concepts such as empathy, emotional labor, and relational care. We aim to disentangle these concepts and provide a more precise definition of compassion that can be applied across different caregiving contexts. This will enable researchers and practitioners to better understand the specific role of compassion in dementia caregiving and guide interventions aimed at enhancing compassionate care.

Furthermore, the inclusion of the CFCF [15] in our review offers a structured lens through which to analyze compassion in dementia caregiving. This framework emphasizes the importance of compassion as a core value in caregiving and provides a comprehensive approach to understanding its various dimensions, such as compassionate design, education, and practice [15]. Using this framework, we will examine how compassion is defined and operationalized across different caregiving settings and identify potential gaps in current practice. Importantly, if findings emerge that do not align with the framework, we will be able to reflect on its appropriateness, limitations and expansion within the context of dementia caregiving. The involvement of patient and caregiving partners as well as knowledge users throughout the review will help ensure the analysis remains grounded in caregiver experiences, while enhancing the relevance and applicability of the findings for both academic and caregiving communities.

### Limitations

There are several methodological limitations that should be acknowledged. First the review will only include studies that can be translated to English, which may lead to language bias and exclude valuable insights from non-English-speaking contexts. A limitation of using machine translation like DeepL is that it may not capture nuanced or technical language accurately, potentially leading to misinterpretation of study details. This could limit the scope of the review, particularly regarding studies from non-English-speaking countries or regions where caregiving practices and cultural conceptualizations of compassion may differ. The inclusion of only peer-reviewed studies also presents a limitation. Although peer-reviewed literature is often considered more methodologically rigorous, it may exclude relevant findings from grey literature such as conference abstracts, reports, and policy documents, which could contain useful information about compassion in caregiving. Grey literature often captures innovative practices, culturally specific approaches, and emerging interventions that may not yet appear in academic publications. To partially mitigate this, reference lists of systematic and scoping reviews will be screened to identify additional relevant primary studies. The exclusion of grey literature will be explicitly considered when interpreting findings and making recommendations for research, policy, and practice. Despite efforts to ensure a comprehensive search, we cannot guarantee that all studies relevant to the topic will be identified. Additionally, variations in how compassion is conceptualized and operationalized across studies could introduce heterogeneity in the findings, making it challenging to draw unified conclusions.

## Conclusion

This scoping review protocol provides an overview of the methodology and methods for a forthcoming scoping review that aims to map and synthesize empirical studies on the conceptualizations, definitions, and operationalization of compassion in dementia caregiving. By exploring how compassion is conceptualized, defined, perceived, and operationalized in caregiving contexts, this review will offer a deeper understanding of the multifaceted role of compassion in dementia care. Through the integration of the CFCF [15], the review will assess compassion across three key areas: design, education, and practice, thereby providing a comprehensive lens to inform future caregiving strategies and interventions. The active involvement of patient and caregiving partners as well as knowledge users ensures that the findings will be relevant to both academic audiences and the broader caregiving community. By synthesizing the diverse perspectives on compassion, this review will contribute to the development of clearer definitions, practical frameworks, and actionable recommendations that can enhance caregiving practices and improve the well-being of family caregivers and care recipients.

## Supporting information

S1 File PRISMA-P-checklist. (PDF)
